# Synthesis of green zinc‐oxide nanoparticles and its dose‐dependent beneficial effect on spermatozoa during preservation: sperm functional integrity, fertility and antimicrobial activity

**DOI:** 10.3389/fbioe.2024.1326143

**Published:** 2024-02-23

**Authors:** Meiaishan Eliezer Lyngdoh, Jyoti Chettri, Vivian F. Kharchandy, Rishav Sheel, Arnab Roy Choudhury, Biplab Sarkar, Arunava Pattanayak, Sourabh Deori, Sayed Nabil Abedin, G. Kadirvel

**Affiliations:** ^1^ Reproduction Biology Laboratory, ICAR Research Complex for NEH Region, Umiam, India; ^2^ ICAR- Indian Institute of Agricultural Biotechnology, Ranchi, India

**Keywords:** antibacterial, anti‐oxidant, artificial insemination, cytotoxicity, fertility, liquid preservation, zinc oxide nanoparticles (ZnO‐NPs)

## Abstract

**Introduction:** The development of an effective extender is important for semen preservation and the artificial insemination (AI) industry. This study demonstrates the beneficial effect of zinc oxide nanoparticles (ZnO-NPs) as an additive to semen extenders to improve semen quality, fertility, and antibacterial activity during liquid preservation in a boar model.

**Methods:** Initially, to find out the safe concentration of ZnO-NPs in sperm cells, a wide range of ZnO-NP concentrations (0, 5, 10, 50, 100, 500, and 1,000 μM) were co-incubated with sperm at 37°C for a cytotoxic study. These NP concentrations were compared to their salt control zinc acetate (ZA) at the same concentrations and to a control group. The effect of the different concentrations of ZnO-NPs on sperm motility, membrane integrity, mitochondrial membrane potential (MMP), and apoptosis was assessed. Accordingly, the non-toxic dose was selected and supplemented in MODENA extender to determine its beneficial effect on the boar semen parameters mentioned and the lipid peroxidation (LPO) levels during liquid preservation at 16°C for 6 days. The non-cytotoxic dosage was subsequently chosen for AI, fertility investigations, and the evaluation of the antibacterial efficacy of ZnO-NPs during preservation hours. An antibacterial study of ZnO-NPs and its salt control at doses of 10 μM and 50 μM was carried out by the colony forming unit (CFU) method.

**Results and discussion:** The cytotoxic study revealed that 5, 10, and 50 μM of ZnO-NPs are safe. Consequently, semen preserved in the MODENA extender, incorporating the non-toxic dose, exhibited 10 and 50 μM ZnO-NPs as the optimal concentrations for beneficial outcomes during liquid preservation at 16°C. ZnO-NPs of 10 μM concentration resulted in a significantly (*p* < 0.05) improved conception rate of 86.95% compared to the control of 73.13%. ZnO-NPs of 10 and 50 μM concentrations exhibit potent antimicrobial action by reducing the number of colonies formed with days of preservation in comparison to the negative control. The investigation concluded that the incorporation of 10 μM ZnO-NPs led to enhancements in sperm motility, membrane integrity, and MMP, attributed to a reduction in the malondialdehyde (MDA) levels. This improvement was accompanied by a concurrent increase in fertility rates, including farrowing rate and litter size, during the liquid preservation process. Furthermore, ZnO-NPs exhibited an antimicrobial effect, resulting in decreased bacterial growth while preserving boar semen at 16°C for 6 days. These findings suggest that ZnO-NPs could serve as a viable alternative to antibiotics, potentially mitigating antibiotic resistance concerns within the food chain.

## 1 Introduction

Artificial insemination (AI) is a simple and cost-effective tool for the rapid dissemination of superior germplasm in the livestock industry. AI in pigs is practiced worldwide to propagate elite genes, reduce labor costs, prevent sexually transmitted diseases, and improve farm economics (Knox, 2016). AI is used in South Asian countries to upgrade nondescript pigs with superior germplasm to enhance productivity in backyard smallholder pig production systems (Govindasamy et al., 2016). Approximately 5% of the population is covered by AI in the world, with an increasing trend every year, in which 99% of pigs are covered using liquid semen preserved at 16°C and the remaining 1% of pigs are inseminated using cryopreserved semen (Johnson et al., 2000). However, the liquid-preserved semen could be stored for a short period of 3–5 days and utilized for AI. The sperm motility, fertility, and litter size were reduced when semen was stored for more than 5 days (Johnson et al., 2000; van den Berg et al., 2014). Therefore, there is a need to develop new and efficient extenders or add new additives to increase the storage period of liquid semen without compromising fertility. Many studies that have been carried out on the beneficial effect of the addition of enzymatic and non-enzymatic additives, membrane stabilizers, and antioxidants in the extender showed improved motility and fertility during fertilization (Murasing et al., 2020). Recently, nanoparticles (NPs) have been utilized in reproductive biology, specifically in sperm biology (Feugang, 2017), because NPs (1–100 nm) are well known for their interactions with biological systems (Lee et al., 2007; Wang, 2008) and their nano-size provides greater surface area and enhanced bioavailability compared to natural salts (Thorek and Tsourkas, 2008). Recent studies demonstrated the positive effect of NPs such as curcumin NPs, Vit E nanoemulsions, and Zn-NPs on sperm attributes during preservation in different species such as camels, rabbits, and bulls (Abdelnour et al., 2020; Sánchez-Rubio et al., 2020; Shahin et al., 2020; Jahanbin et al., 2021).

Bacterial contamination in semen is known to affect sperm quality during preservation, and it causes infection in the female reproductive tract, leading to reduced fertility. Various antibiotics such as gentamycin and streptomycin–penicillin have been used to inhibit bacterial growth during semen preservation. Nevertheless, there has been a growing frequency of observations regarding the escalating resistance of microorganisms to various antibiotics in the food chain (Santos et al., 2021). Examinations into metal oxide NPs, including silver (Ag), iron oxide (FeO), titanium oxide (TiO2), copper oxide (CuO), and zinc oxide (ZnO), have revealed their robust antibacterial effects, making them a potential substitute for antibiotics. Zinc oxide nanoparticles (ZnO-NPs) have been researched and utilized across various domains such as biomedical sciences, agriculture, food processing, drug delivery, and cancer therapy (Mishra et al., 2017). In recent times, several studies have showcased the encouraging potential of NPs as efficient antimicrobial agents, offering an alternative to antibiotics. This is particularly evident in examinations of toxicity and antimicrobial effects related to AgNPs in swine sperm (Pérez-Duran et al., 2020; Sánchez-López et al., 2020).

Previous studies of physically or chemically synthesized ZnO-NPs on ram semen (Heidari et al., 2018) and bull semen (Jahanbin et al., 2021) exhibited a positive role of the NP acting as an effective antioxidant in improving the post-thaw sperm quality after cryopreservation. However, physically and chemically synthesized NPs exhibit side effects on the biological system. Therefore, green NPs or NPs of biological origin are required (Donga et al., 2020). Therefore, the current study focused on investigating the effect of biologically synthesized and characterized green ZnO-NPs on boar sperm quality and fertility. Furthermore, the ongoing study delved into the antimicrobial properties of ZnO-NPs with the objective of diminishing the need for conventional antibiotics in the preservation and processing of sperm for AI.

## 2 Materials and methods

### 2.1 Procurement of chemicals and reagents

Reagents for preparing Sperm Tyrode’s albumin lactate pyruvate (Sp-TALP) media and MODENA extender, 2-thiobarbituric acid (TBA), trichloroacetic acid (TCA), hydrochloric acid (Finar Reagent), and Dulbecco’s phosphate-buffered saline (DPBS; 10X), were procured from HiMedia Laboratories Pvt. Ltd., Maharashtra, India, and Merck Ltd., Darmstadt, Germany. Fluorescent dyes such as 5(6)-carboxyfluorescein diacetate (CFDA) and propidium iodide (PI) (≥94%) were purchased from Sigma-Aldrich Chemicals Private Limited, Bangalore, India, and 5,5′,6,6′-tetrachloro-1,1′,3,3′-tetraethylbenzimidazolocarbocyanine iodide (JC-1) was purchased from Morek Life Solanos Pvt. Ltd., Maharashtra, India, for the semen quality assays. ZnO-NPs and zinc acetate (ZA) were obtained from ICAR-Indian Institute of Agricultural Biotechnology, Ranchi, India. Zinc acetate dihydrate and sodium hydroxide for NP synthesis and reagents of analytical purity grade were commercially purchased from Merck Ltd., Darmstadt, Germany. For antimicrobial studies, Plate Count Agar (PCA) and sterile disposable Petri plates (90 mm × 15 mm) were purchased from HiMedia Laboratories Pvt. Ltd., Maharashtra, India.

### 2.2 Preparation of different media and extenders for sperm processing

Sperm Tyrode’s albumin lactate pyruvate (Sp‐TALP) media were prepared by dissolving the following: 100 mM NaCl, 3.1 mM KCl, 25 mM NaHCO_3_, 0.29 mM NaH_2_PO_4_, 21.6 mM C_3_H_5_NaO_3_, 2.0 mM CaCl_2_, 1.5 mM MgCl_2_, and 10 mM HEPES (Parrish et al., 1988). MODENA extender for semen extension was prepared with the following: 152.6 mM C_6_H_12_O_6_, 23.46 mM Na_3_C_6_H_5_O_7_.2H_2_O, 6.31 mM C_10_H_14_N_2_Na_2_O_8_.2H_2_O, 11.9 mM NaHCO_3_, 13.8 mM C_6_H_8_O_7_.H_2_O, 46.56 mM C_4_H_11_NO_3_, and strepto-penicillin 1.8 mgf/mL (Estienne et al., 2007).

### 2.3 Preparation of the nanomaterial

The chemical precipitation method was followed for ZnO-NP synthesis. In brief, a 50 mL solution of ZA dihydrate (20 mM) was prepared and placed on a magnetic stirrer for 20 min for proper mixing. Sodium hydroxide (0.1 M) solution was added dropwise to this solution until the pH became 9 to 10. Then, the solution was stirred at 750–1,000 rpm for 2 h. During this time, a white-colored precipitate of zinc hydroxide was formed. The precipitate was centrifuged at 4,000 rpm for 20 min followed by repeated washing with distilled water and ethanol to remove trace impurities. The precipitate was dried at 105°C in a hot-air oven overnight to obtain ZnO-NPs. For encapsulation, the synthesized ZnO-NPs were added slowly into a 0.05% solution with a suitable encapsulation material in a 1:25 w/v ratio. The solution of carboxymethyl cellulose (CMC) with ZnO-NPs was placed on a magnetic stirrer for 2 h for the encapsulation of ZnO-NPs with CMC. The ZnO suspension was centrifuged at 5,000 rpm for 10 min, followed by repeated washing with distilled water and ethanol to remove extra CMC. The final precipitate was dried at 60–80°C in a hot-air oven overnight to obtain CMC-encapsulated ZnO-NPs.

### 2.4 Characterization of the nanomaterial

Synthesized ZnO-NPs were characterized by using a UV–visible spectrophotometer (CECIL CE 7200, UK) at a 200 nm–700 nm range. To evaluate the potential functional groups associated with synthetic methods, Fourier-transform infrared spectroscopy (FT-IR) analysis was conducted (Nicolet iS5 FT-IR spectrometer, Thermo Fisher Scientific, United States) in the range of 400–4,000 cm^−1^ at a resolution of 4 cm^−1^. A small amount of ZnO nano-powder was taken for KBr pellet preparation and, thereafter, was processed for FT-IR study and analyzed through inbuilt software applications. X-ray diffraction (XRD) was performed (SmartLab 9 kW Rigaku, Japan, X-ray diffractometer). The inbuilt software program was used for the assignment of reflections and analysis of the XRD patterns. The surface morphology of the synthesized ZnO-NPs was characterized by field emission scanning electron microscopy (FESEM, Carl Zeiss Sigma 300, Germany) combined with focused ion beams. The energy-dispersive X-ray spectroscopy (EDS) for the elemental analysis of the synthesized NPs was also carried out using the same instrument and in a particular area of the samples.

### 2.5 Boar management and semen collection

Six adult healthy crossbreed boars (75% Hampshire X 25% Niang Megha inheritance) were selected for the study. The boars were maintained in pig breeding farms of the institute and routinely used for semen collection and AI purposes. All boars were kept in accordance with the standard management practices in a pen system for housing. They were fed standard concentrated mash feed twice daily, and water was provided *ad libitum*. The experiment was approved by the Institutional Animal Ethics Committee (RC/IAEC/2020/2). Semen was collected by the gloved-hand method twice weekly from each boar inside pre-warmed (38°C) collection bags fitted with filters and placed inside semen collection cups (Minitube, Germany). The collected semen was evaluated for motility and concentration, and samples with more than 70% sperm progressive motility and more than 200 million sperm per mL concentration were processed further for all the experiments.

### 2.6 Assessment of the safe range of ZnO-NP concentration on sperm cells

The experiment aimed to determine the safe concentrations of ZnO-NPs that cause no adverse cytotoxic effect on boar sperm. Different concentrations of ZnO-NPs, *viz.*, 5 μM, 10 μM, 50 μM, 100 μM, 500 μM, and 1,000 μM, and the same concentrations for ZA as a salt control were selected and dissolved in Sp-TALP media (Galatino-Homer et al., 1997). The Sp-TALP media containing no ZnO-NP/no ZA were taken as a control. A total of 42 semen ejaculates, seven ejaculates from each boar, were utilized for the study. Semen samples were diluted in 1:3 dilutions in media containing 40 million sperm cells/mL in 2 mL aliquots using a split sample technique for each treatment. Samples were incubated for 0 h and 1 h in a CO_2_ incubator (Thermo Fisher Scientific, United States) with 5% CO_2_ and 95% humidity at 37°C and assessed for cytotoxic effect on the different sperm functional attributes such as motility, plasma membrane integrity, mitochondrial membrane potential (MMP), and apoptosis.

### 2.7 Effect of ZnO-NPs on sperm quality parameters during liquid preservation

Based on the previous experiment, safe concentrations of ZnO-NPs with insignificant cytotoxic effects on boar sperm cells were selected for studying their beneficial effect on the liquid preservation of boar semen for a short duration. Accordingly, MODENA extender containing 5 μM, 10 μM, and 50 μM of ZnO-NPs and the same concentration of ZA as a salt control was utilized for the study. MODENA extender containing no ZnO-NPs or ZA was kept as a control. A total of 50 semen ejaculates were collected and diluted 1:3 with MODENA extender containing different concentrations of NPs and ZA to a final concentration of 40 million sperm cells/mL in 2 mL aliquots, using the split sample technique. The semen samples were then stored in a BOD incubator (Narang Scientific Works Pvt. Ltd, New Delhi, India) at 16°C for 6 days and evaluated for sperm motility, viability, plasma membrane integrity, MMP, and lipid peroxidation (LPO) by MDA assay at day 0, day 3, and day 5.

### 2.8 Assessment of sperm functional attributes

#### 2.8.1 Sperm motility

Sperm progressive motility (%) was measured subjectively by the wet film method taking a drop of 10–15 µL of semen on a pre-warmed glass slide, covering it with a cover slip, and then, examining it under a phase-contrast microscope (Olympus, BX51 FT, Japan) at ×400 magnification. At least ten widely spaced fields were examined to provide an estimate of the motile spermatozoa.

#### 2.8.2 Sperm viability, membrane integrity, and MMP

Sperm viability and membrane integrity (%) were assessed using the dual fluorescent dyes CFDA and PI staining with slight modifications (Garner et al., 1986), while the MMP was assessed using JC-1 fluorescent dye (Garner et al., 1997). Approximately 10 million sperm cells were washed once using pre-warmed PBS (1X) by centrifugation at 500 *g* for 5 min and re-suspended in 200 μL PBS. For the sperm viability and membrane integrity test, 2 μL of CFDA (from stock solution 4 mg/mL in DMSO) was added to 200 μL sperm suspension and incubated in the dark at 37°C for 5 min; 4 μL of PI (stock solution 0.5 mg/mL in PBS) was then added to the same sperm suspension and incubated in the dark at 37°C for 5 min. For the determination of sperm MMP, 5 μL of the working solution of JC-1 (0.153 mM in PBS; Sigma-Aldrich, United States) was added into the same sperm suspension of 200 μL and incubated in the dark at 37°C for 5 min. Following incubation, the pre-stained sperm suspension was washed using pre-warmed PBS by centrifugation at 500 *g* for 5 min and re-suspended in 100 μL of PBS. A 10–15 μL drop of the cell suspension was placed on a clean glass slide, covered with a cover slip, and examined under a fluorescent microscope (Eclipse 80i, Nikon, Japan) at ×20 magnification. The cell emitting green fluorescence was viewed in FITC (EX 462–495, BA 515–555), and those with red fluorescence were viewed in TRITC (EX 540/25, BA 605/55). A total of 200 fluorescent-tagged sperm cells were examined, and CFDA- and PI-stained cells were classified into the following: a) live and intact cells, cells emitting green fluorescence at the head region; b) dead and damaged cells, red fluorescence at the head; and c) morbid cells, emitting both green and red fluorescence in the head region. Similarly, a total of 200 fluorescent-tagged cells were examined for JC-1 staining and classified accordingly as high MMP, red–orange fluorescence at the mid-piece, and low MMP, green fluorescence at the mid-piece.

### 2.9 Apoptotic assay

The Annexin-V-FLUOS Staining Kit (11858777001; Roche) was used for this assay. After washing with PBS, cells were re-suspended in 100 μL of Annexin-V-FLUOS labeling solution and then incubated for 15 min at room temperature in the dark. A 10–15 μL drop of the cell suspension was placed on a clean glass slide, covered with a cover slip, and examined under a fluorescent microscope at ×20 magnification. A total of 200 fluorescent-tagged sperm cells were counted according to the fluorescence emitted, and the cell population was classified as follows: apoptotic cells, green fluorescent; necrotic cells, green and red fluorescent; dead cells, red fluorescent; and live cells, non-fluorescent.

### 2.10 Lipid peroxidation assay

LPO assay was performed using TBA, as described by Buege and Aust (1978) and further modified by Suleiman et al. (1996). A total of 15 g of TCA and 0.375 g of TBA were dissolved in 100 mL of 0.25 N HCl. Approximately 10 million sperm cells were washed once using pre-warmed PBS by centrifugation at 500 *g* for 5 min and re-suspended in 1 mL PBS. An amount of 2 mL of the TBA–TCA reagent was added, and the mixture was boiled for 15 min and allowed to cool. The supernatant was then separated by centrifugation at 1,500 *g* for 15 min. The absorbance of the supernatant was measured at 535 nm, and the MDA concentration was determined by the specific absorbance coefficient (1.56 × 10^5^/mol/cm^3^).

$$\text{MDA produced }\left(\mu M/\text{ml}\right)=\left\{O.D.\times 10^{6}\times \text{total volume }\left(3\text{ ml}\right)\right\}/\;\left\{1.56\times 10^{5}\times \text{test volume }\left(1\text{ ml}\right)\right\}=\left(O.D.\;X\;30\right)/\;1.56.$$

### 2.11 Artificial insemination and the assessment of fertility

An amount of 80 mL of semen diluted in MODENA extender containing 10 μM ZnO-NPs was packed in 95 mL QuickTip Flexitube^®^ (Minitube, Germany) and sealed using a sealing machine (Minitüb Gmbh, Germany). A total of 300 estrus sows/gilts in standing heat were artificially inseminated using a Foamtip Safelock^®^ porcine insemination catheter (Minitube, Germany). A total of 150 AIs were performed with MODENA containing 10 μM ZnO-NPs, out of which 50 AIs were carried out with day 0 semen, 50 AIs were carried out with day 3 semen, and 50 AIs were carried out with day 5 semen. A total of 150 AIs were performed using the control or MODENA containing no ZnO-NPs and no ZA, out of which 50 AIs were carried out with day 0 semen, 50 AIs were carried out with day 3 semen, and 50 AIs were carried out with day 5 semen. Following insemination, after 6 weeks, pregnancy diagnosis was carried out in non-cycling gilts/sows by the Doppler method utilizing a trans-abdominal probe (EXAGO, Asha Medical and Co., New Delhi, India). After farrowing, the rate of farrowing and size of litter at birth were calculated.

### 2.12 Assessment of the antibacterial activity of ZnO-NPs

The bacterial load in the semen sample was estimated using the standard plate count method (Tsakmakidis et al., 2020). The experiment was carried out for semen media containing ZnO-NPs with concentrations of 10 μM and 50 μM and the same concentrations for ZA as a salt control and compared to their positive control (media containing 1.8 mg/mL of the antibiotic strepto-penicillin and no NP or salt control) and negative control (semen media containing no antibiotic nor NP/salt control). Extended semen samples were diluted up to 10^6^ dilutions with 1X PBS, and 100 µL of each extended diluted sample was spread into PCA and incubated in a CO_2_ incubator with 5% CO_2_ and 95% humidity at 37°C. Two replica plating for each dilution per concentration was carried out. The plates were observed after 24 h and 48 h for colony formation, and colonies were manually counted and recorded as colony forming units (CFUs) per mL. The CFU was calculated using the following formula:

$$\text{CFUs}/\text{mL}=\frac{\text{Number}\;\text{of}\;\text{colonies}\;X\;\text{dilution}\;\text{factor}}{\text{Amount}\;\text{plated}}.$$

### 2.13 Statistical analysis

The datasets generated for this study were in mean ± SE, and significant tests were performed using IBM SPSS Statistics 23 software application. A generalized linear model for univariate analysis was carried out. A *post hoc* test for the treatment of ZnO-NP and ZA salt compared to the control was conducted. The variance was analyzed using the Tukey and Duncan tools, and *p*-value<0.05 was chosen as the significance level for all tests. The conception rate was displayed in percentage and significance level at (χ^2^ *p*-value <0.05). For pregnancy and farrowing rates, the Kruskal–Wallis H test was carried out with binary data for significant differences among the treatment groups. Duncan’s multiple range test was performed to make all pairwise comparisons among the parameters wherever a significant difference was obtained. For the antibacterial activity assay, CFUs/mL with zero colonies were positioned at value 0 along the *X*-axis; for CFUs/mL ranging from 1–99 colonies, they were positioned at 10^1^, and other CFUs/mL were positioned according to their range in CFUs/mL along the *x*-axis. The datasets generated for evaluating the antibacterial activity underwent a check for sphericity assumptions using Mauchly’s test to examine the within-subject effects. To analyze antibacterial activity across various preservation times, repeated measures ANOVA was employed to draw statistical conclusions. All treatments were compared to the control to draw valid conclusions.

## 3 Results

### 3.1 Characterization of the synthesized zinc oxide nanomaterial

**FIGURE 1:**
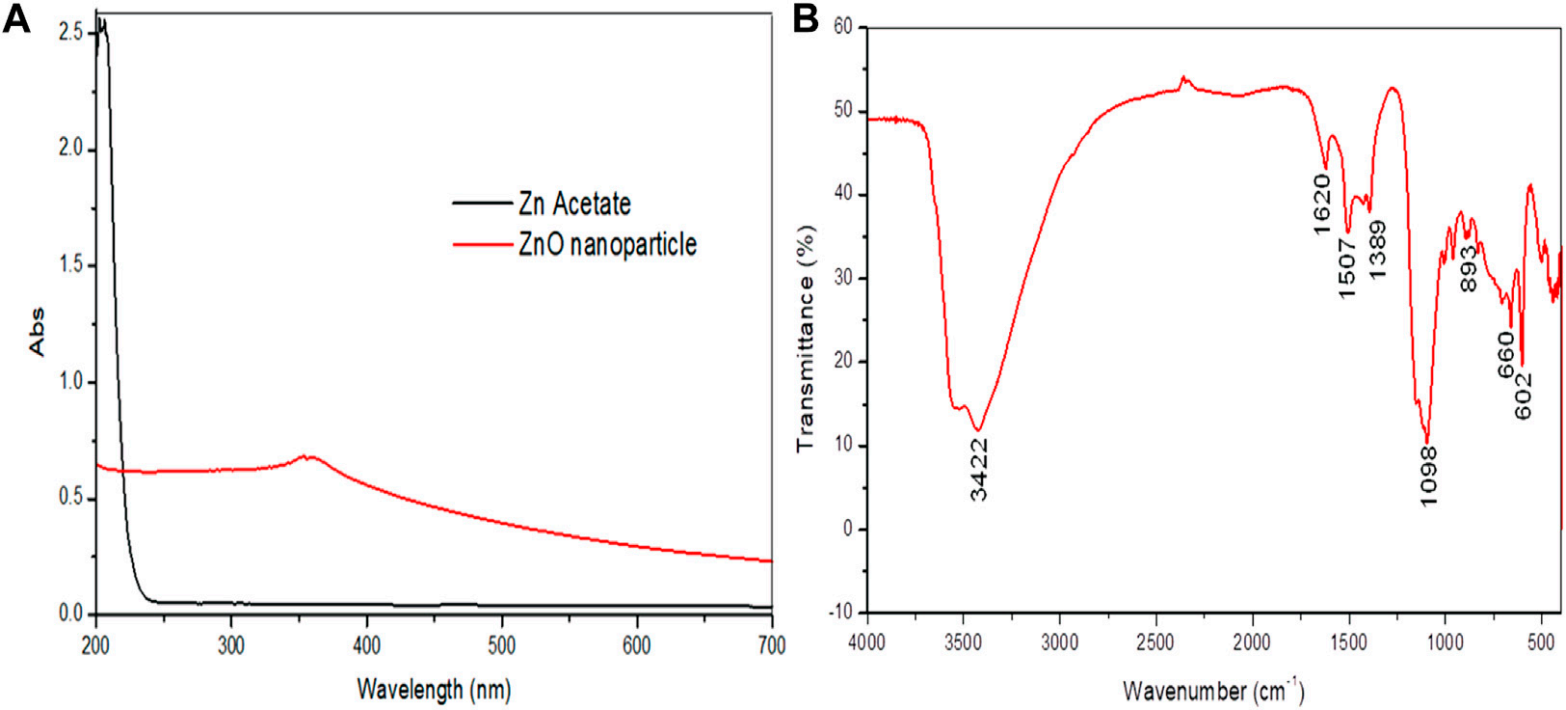
Synthesized zinc oxide nanoparticles. **(A)** UV–visible spectra of the synthesized zinc oxide nanoparticles and zinc acetate showing peak absorbance. **(B)** FT-IR spectra of the synthesized zinc oxide nanoparticles.

**FIGURE 2:**
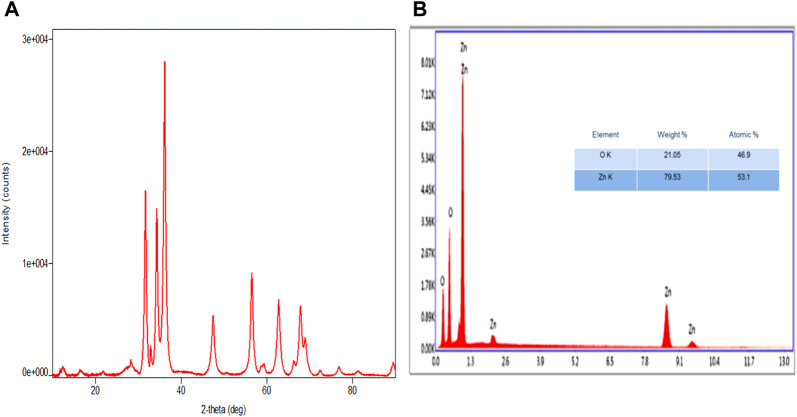
Synthesized zinc oxide nanoparticles. **(A)** XRD spectra pattern of zinc oxide nanoparticles. **(B)** EDX spectra of zinc oxide nanoparticles.

**FIGURE 3:**
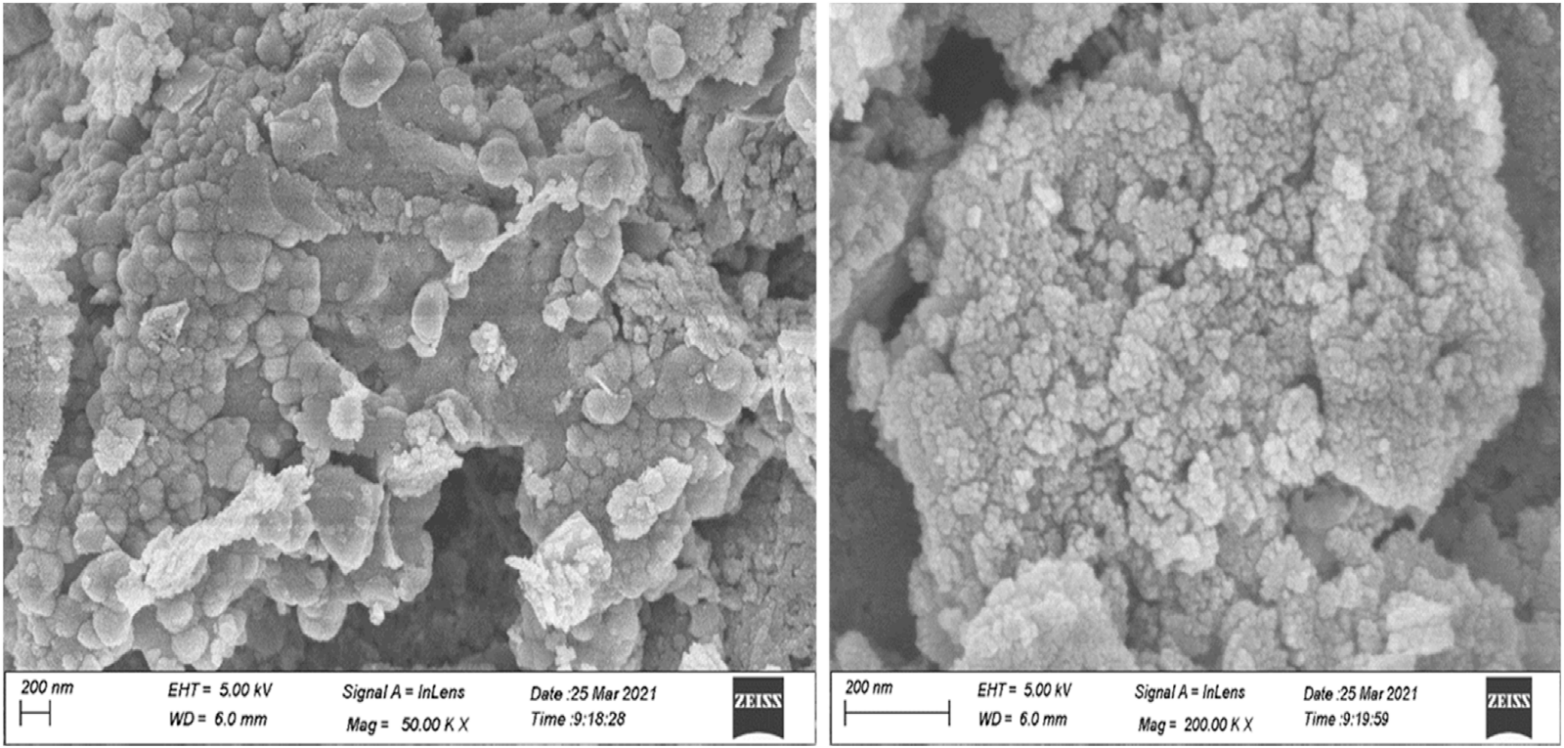
FESEM images of the synthesized zinc oxide nanoparticles at different magnifications.

UV–visible spectra exhibited peaks near 350 nm conforming to plasmon-resonance absorption in ZnO-NPs (Figure 1A). The FT-IR spectra of the synthesized ZnO-NP showed peaks at 602 cm^−1^ and 660 cm^−1^ for the ZnO absorption band and confirmed the variation in the banding pattern during its synthesis. The peaks observed at 1620 cm^−1^,1507 cm^−1^, and 1389 cm^−1^ indicate the presence of –OH and C=O residues, which may be due to ZA precursors used in the reaction (Figure 1B). The XRD analysis showed that the prominent diffraction peaks located at 31.7^o^, 34.3^o^, 36.2^o^, 47.4^o^, 56.5^o^, 62.6^o^, 67.7^o^, and 68.8^o^ have been indexed as hexagonal wurtzite phases of ZnO-NPs (Figure 2A). The Joint Committee on Powder Diffraction Standards (JCPDS) data (JCPDS Card No. 36–1451) of XRD analysis also substantiated the synthesis of ZnO-NPs. The EDS analysis of the ZnO-NP for the selected area confirmed the presence of the Zn, which is depicted in Figure 2B. The measured mean particle size was 74.59 ± 17.47 nm (mean ± SD; Supplementary Figure S1). The surface morphological analysis of the synthesized ZnO-NP was carried out by FESEM, which is depicted in Figure 3. The ZnO-NP showed a granular structure and also showed a rough surface overview at a higher magnification.

### 3.2 Assessment of the safe range of ZnO-NP concentrations on sperm cells

The study recorded no significant change (*p* > 0.05) in sperm progressive motility between the control and the different concentrations of ZnO-NPs and ZA (5, 10, 50, and 100 μM) at both 0 h and 1 h. However, a significant (*p* < 0.05) reduction was observed in sperm progressive motility at concentrations of 500 μM and 1,000 μM for both ZnO-NPs and ZA salt compared to the control at both 0 h and 1 h (Table 1). Similarly, the viability and membrane integrity showed no significant (*p* > 0.05) differences when comparing all concentrations of ZnO-NPs and ZA to the control at 0 h. However, at 1 h incubation, there was a significant (*p* < 0.05) increase in viability and membrane integrity with 10 μM and 50 μM ZnO-NP concentrations, whereas 500 μM ZA concentration significantly (*p* < 0.05) decreased these parameters compared to the control (Table 2). Consequently, a significant (*p* < 0.05) elevation in high mitochondrial membrane potential (H-MMP) within sperm cells was observed at ZnO-NP concentrations of 10 μM and 50 μM. Conversely, concentrations of ZA at 500 μM and 1,000 μM significantly (*p* < 0.05) reduced the H-MMP of sperm cells compared to the control at 1 h of preservation (Table 2). In the ZnO-NP treatment groups, a concentration of 500 μM significantly (*p* < 0.05) decreased the sperm viability and membrane integrity compared to lower concentrations (5, 10, 50, and 100 μM) at 0 h. A similar pattern was observed after 1 h of incubation, where the higher concentration (500 μM) significantly (*p* < 0.05) reduced the viability and membrane integrity compared to the lower concentrations. Regarding sperm MMP, there were no significant (*p* > 0.05) changes at all incubated concentrations at 0 h, but at 1 h, MMP was significantly (*p* < 0.05) reduced at the highest concentration (1,000 μM)

**TABLE 1:** Cytotoxic effect of different concentrations of ZnO-NPs and ZA on sperm progressive motility (%).

Concentrations (μM)	ZnO-NPs		ZA		Control	
	0 h	1 h	0 h	1 h	0 h	1 h
5	84.33 ± 1.51^a^	75 ± 1.66^a^	84.33 ± 1.51^a^	75.25 ± 1.75^a^	84.33 ± 1.51^a^	71 ± 1.99^ab^
10	84.33 ± 1.51^a^	75.75 ± 1.63^a^	84.33 ± 1.51^a^	75 ± 1.77^a^		
50	84.33 ± 1.51^a^	74.5 ± 1.84^ab^	84.33 ± 1.51^a^	72.25 ± 1.97^ab^		
100	83.66 ± 1.42^a^	67.25 ± 1.48^abc^	83.66 ± 1.42^a^	66 ± 1.93^bc^		
500	75.66 ± 1.59^b*^	61.75 ± 1.63^cd*^	76.33 ± 1.33^b*^	60.25 ± 1.90^cd*^		
1000	73.00 ± 1.55^c*^	54.25 ± 1.84^d*^	72.66 ± 1.24^c*^	54.25 ± 2.19^d*^		

Data are shown as mean ± SE; values with different superscripts in each time (h) differ significantly (*p* ≤ 0.05).

*Significant (*p* < 0.05).

**TABLE 2:** Effect of different concentrations of ZnO-NPs and ZA on live and intact sperm (%) and mitochondrial membrane potential (%).

Concentration (μM)	Live and intact (%)						High mitochondrial membrane potential (H-MMP) (%)					
	ZnO-NPs		ZA		Control		ZnO-NPs		ZA		Control	
	0 h	1 h	0 h	1 h	0 h	1 h	0 h	1 h	0 h	1 h	0 h	1 h
5	81.81 ± 1.22^abc^	78.22 ± 0.83^ab^	79.73 ± 0.84^abcde^	76.53 ± 0.76^bc^	80.78 ± 0.55^abcd^	75.95 ± 0.48^bcd^	81.64 ± 1.45^abc^	77.89 ± 0.77^bcd^	83.77 ± 1.45^abc^	80.01 ± 0.77^ab*^	81.08 ± 1.21^abc^	77.37 ± 0.67^cde^
10	82.84 ± 1.05^a^	81.73 ± 0.71^a*^	80.71 ± 0.73^abcde^	75.61 ± 1.00^bcd^			85.07 ± 1.62^a^	81.32 ± 0.86^a*^	82.44 ± 1.28^ab^	78.69 ± 0.69^bc^		
50	82.41 ± 0.95^ab^	80.45 ± 0.82^a*^	81.04 ± 0.98^abcd^	74.94 ± 0.76^bcd^			83.86 ± 1.60^ab^	80.11 ± 0.84^ab*^	81.05 ± 1.20^abc^	77.30 ± 0.66^cde^		
100	80.47 ± 0.79^abcde^	75.97 ± 0.60^bcd^	79.31 ± 0.78^abcde^	72.38 ± 0.64^de^			82.54 ± 1.72^abc^	78.79 ± 0.89^bc^	78.89 ± 1.17^abc^	75.14 ± 0.63^ef^		
500	78.32 ± 0.91^bcde^	72.87 ± 0.79^cd^	77.68 ± 0.75^cde^	70.17 ± 0.75^ef*^			80.91 ± 1.68^abc^	77.15 ± 0.89^cde^	77.38 ± 1.08^bc^	73.63 ± 0.58^fg*^		
1000	-	-	-	-			79.52 ± 1.78^abc^	75.77 ± 0.94^def^	76.00 ± 1.13^c^	72.25 ± 0.60^g*^		

Data are shown as mean ± SE; values with different superscripts in each time (h) differ significantly (*p* ≤ 0.05).

*Significant (*p* < 0.05).

The apoptotic assay recorded a non-significant (*p* > 0.05) change in the percent of apoptotic sperm cells at different concentrations of both ZnO-NPs and ZA in comparison to the control at 0 h. However, significantly (*p* < 0.05) lower apoptotic sperms were recorded with ZnO-NPs of 5–10 μM, while at a concentration of 500–1,000 μM, apoptosis increased significantly (*p* < 0.05) at both 0 and 1 h of preservation. On the other hand, ZA at 100 μM and above increased apoptosis to significant levels (*p* < 0.05) compared to the control at 1 h (Table 3).

**TABLE 3:** Effect of different concentrations of ZnO-NPs and ZA on apoptosis and necrosis of sperm cells (%).

Concentration (μM)	Apoptotic (%)					
	ZnO-NPs		ZA		Control	
	0 h	1 h	0 h	1 h	0 h	1 h
5	1.58 ± 0.20^b^	2.98 ± 0.27^d^	2.37 ± 0.49^ab^	4.77 ± 0.38^cd^	2.65 ± 0.29^ab^	5.12 ± 0.29^cd^
10	1.53 ± 0.26^b^	3.37 ± 0.36^d^	2.43 ± 0.17^ab^	6.21 ± 0.57^bc^		
50	2.21 ± 0.35^ab^	5.92 ± 3.01^bc^	2.98 ± 0.34^a^	6.48 ± 0.48^bc^		
100	3.02 ± 0.50^a^	5.93 ± 0.48^bc^	2.51 ± 0.39^ab^	8.06 ± 0.46^ab*^		
500	3.12 ± 0.64^a^	7.13 ± 0.34^abc^	2.37 ± 0.22^ab^	9.01 ± 0.36^a*^		
1000	3.58 ± 0.77^a^	8.39 ± 0.31^ab*^	3.26 ± 0.37^a^	6.39 ± 0.25^bc^		

Data are shown as mean ± SE; values with different superscripts in each time (h) differ significantly (*p* ≤ 0.05).

*Significant (*p* < 0.05).

### 3.3 Assessment of the safe dose of ZnO-NPs on boar sperm functional attributes during liquid preservation

**FIGURE 4:**
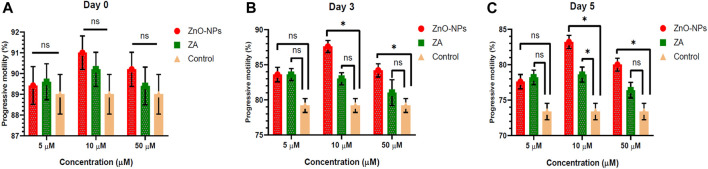
Effect of different concentrations of ZnO-NPs and ZA on sperm progressive motility (%) during liquid preservation of boar semen. **(A)**. Effect of ZnO-NPs and ZA at different concentrations on sperm progressive motility (%) at day 0. **(B)**. Effect of ZnO-NPs and ZA at different concentrations on sperm progressive motility (%) at day 3. **(C)**. Effect of ZnO-NPs and ZA at different concentrations on sperm progressive motility (%) at day 5. “*”: significant (*p* < 0.05) difference in comparison to control; ns: non-significant (*p* > 0.05). ZnO-NPs: zinc oxide nanoparticles; ZA: zinc acetate.

**FIGURE 5:**
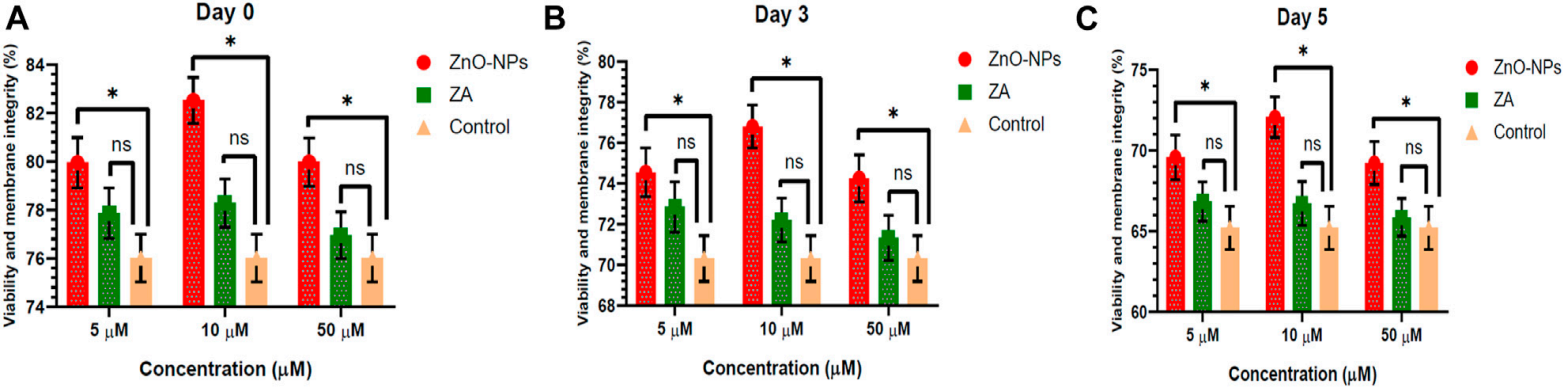
Effect of different concentrations of ZnO-NPs and ZA on sperm viability and membrane integrity (%) during liquid preservation of boar semen. **(A)**. Effect of ZnO-NPs and ZA at different concentrations on sperm viability and membrane integrity (%) at day 0. **(B)**. Effect of ZnO-NPs and ZA at different concentrations on sperm viability and membrane integrity (%) at day 3. **(C)**. Effect of ZnO-NPs and ZA at different concentrations on sperm viability and membrane integrity (%) at day 5. “*”: significant (*p* < 0.05) difference in comparison to control; ns: non-significant (*p* > 0.05). ZnO-NPs: zinc oxide nanoparticles; ZA: zinc acetate.

**FIGURE 6:**
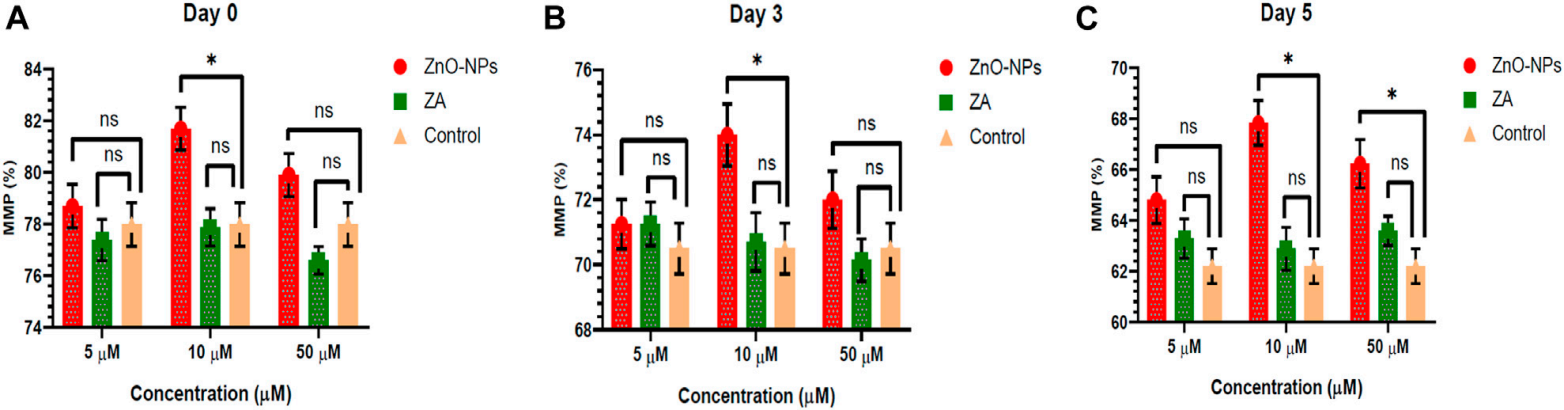
Effects of different concentrations of ZnO-NPs and ZA on sperm mitochondrial membrane potential (%) during liquid preservation of boar semen. **(A)**. Effect of ZnO-NPs and ZA at different concentrations on sperm mitochondrial membrane potential (%) at day 0. **(B)**. Effect of ZnO-NPs and ZA at different concentrations on sperm mitochondrial membrane potential (%) at day 3. **(C)**. Effect of ZnO-NPs and ZA at different concentrations on sperm mitochondrial membrane potential (%) at day 5. “*”: significant (*p* < 0.05) difference in comparison to control; ns: non-significant (*p* > 0.05). ZnO-NPs: zinc oxide nanoparticles; ZA: zinc acetate; and MMP: mitochondrial membrane potential.

Non-cytotoxic doses of ZnO-NPs at 5–50 μM and ZA at equivalent concentrations, when incorporated into the MODENA extender for liquid semen preservation at 16°C, exhibited no significant (p < 0.05) alteration in sperm progressive motility (%) compared to the control on day 0 (Figure 4; Supplementary Table S1). However, progressive motility (%) was significantly (p < 0.05) increased for 10 μM and 50 μM of ZnO-NPs compared to that of the control at day 3 and day 5. Similarly, ZnO-NPs at concentrations of 5–50 μM significantly (p < 0.05) preserved the cell’s viability and membrane integrity on day 0, 3, and 5 in comparison to both ZA and the control (Figure 5; Supplementary Table S2). The sperm MMP during preservation exhibited a significant (p < 0.05) increase at 10 μM ZnO-NPs compared to the control on days 0 and 3. Furthermore, both 10 μM and 50 μM concentration of ZnO-NPs demonstrated significantly (p < 0.05) higher MMP compared to the control on day 5 (Figure 6; Supplementary Table S3).

A significant (*p* < 0.05) reduction in the LPO levels was revealed at 10 μM and 50 μM ZnO-NPs, as well as the ZA salt control, compared to the control on day 3. Additionally, on day 5, both 5–50 μM ZnO-NPs and 10–50 μM ZA exhibited a significant (*p* < 0.05) decrease in the LPO levels in comparison to the control (Table 4). Hence, ZnO-NPs at 10 μM and 50 μM concentrations decreased the LPO of sperm cells that were liquid-preserved at 16°C for up to 6 days. Therefore, 10 μM of ZnO-NP in a lower dose than 50 μM was selected as it possessed a beneficial effect during boar liquid semen preservation at 16°C, improving sperm motility, viability, membrane integrity, and MMP and reducing LPO in cells during storage.

**TABLE 4:** LPO assay by MDA estimation on boar semen during liquid preservation.

MDA (nmol/mL)	Group	Concentration (μM)								
		Day 0			Day 3			Day 5 (^b^)		
		5	10	50	5	10	50	5	10	50
	**ZnO-NPs**	202.7 ± 32.27^a^	163.1 ± 25.77^a^	146.7 ± 24.83^a^	350.6 ± 29.44^ab^	288.3 ± 27.35^a*^	281.7 ± 25.64^a*^	457.3 ± 33.64^a*^	393.7 ± 29.74^a*^	387.5 ± 27.20^a*^
	**ZA**	218.5 ± 39.05^a^	161.3 ± 23.48^a^	169.8 ± 24.73^a^	365.2 ± 39.74^ab^	293.5 ± 25.81^a*^	297.9 ± 25.03^a*^	509.0 ± 43.05^ab^	396.7 ± 29.02^a*^	397.1 ± 30.52^a*^
	**Control**	245.0 ± 36.22^a^			474.8 ± 42.61^b^			636.0 ± 47.64		

Data are shown as mean ± SE; values with different superscripts in each day differ significantly (*p* ≤ 0.05).

*Significant (*p* < 0.05) from control.

The selected dosage of 10 μM ZnO-NPs was supplemented with MODENA extender and tested for farrowing rate, litter size, stillbirth, and weak piglets after AI was performed.

### 3.4 *In vivo* fertility rate after artificial insemination

The *in vivo* fertility experiment recorded that the MODENA extender containing 10 μM ZnO-NPs had a significantly higher farrowing rate on day 3 (χ^2^ *p*-value<0.05) and day 5 (χ2 *p*-value<0.01) of preserved semen compared to the MODENA extender without ZnO-NPs (Table 5). Similarly, the litter size at birth was significantly higher on day 3 (χ2 *p*-value<0.05) and day 5 (χ2 *p*-value<0.01) after AI with the MODENA extender+10 μM ZnO-NPs compared to the MODENA extender without ZnO-NPs. However, the percentage of stillbirth and weak piglets did not differ significantly (*p* > 0.05) between AI with MODENA extender plus ZnO-NPs and only MODENA extender (Table 5).

**TABLE 5:** Effect of MODENA containing 10 μM ZnO-NPs and the control on *in vivo* fertility parameters.

Parameter	MODENA + ZnO-NPs (n = 150 sows)			Control (n = 150 sows)		
	Day 0	Day 3	Day 5	Day 0	Day 3	Day 5
**Farrowing rate (%)**	86 (43)^aa^	82 (41)^ab^	76 (38)^cc*^	84 (42)^ab^	78 (39)^cc*^	70 (35)^dd**^
**Litter size at birth (nos.)**	9.74 ± 0.52^aa^	9.57 ± 0.43^ab^	9.12 ± 0.41^bc*^	9.43 ± 0.35^ab^	9.22 ± 0.56^bc*^	8.7 ± 0.37^dd**^
**Stillbirth (%)**	0.35 ± 0.07^aa^	0.31 ± 0.06^ab^	0.28 ± 0.04^ac^	0.28 ± 0.05^ad^	0.32 ± 0.06^ad^	0.23 ± 0.03^ae^
**Weak piglets (%)**	0.24 ± 0.03^aa^	0.21 ± 0.02^ab^	0.18 ± 0.04^ac^	0.31 ± 0.07^ad^	0.28 ± 0.05^ad^	0.15 ± 0.04^ae^

Values with different superscripts (a, b, c, d, and e) in each day differ significantly (*p* < 0.05).

*Significant (*p* < 0.05); **highly significant (*p* < 0.01).

### 3.5 Antimicrobial activity

**FIGURE 7:**
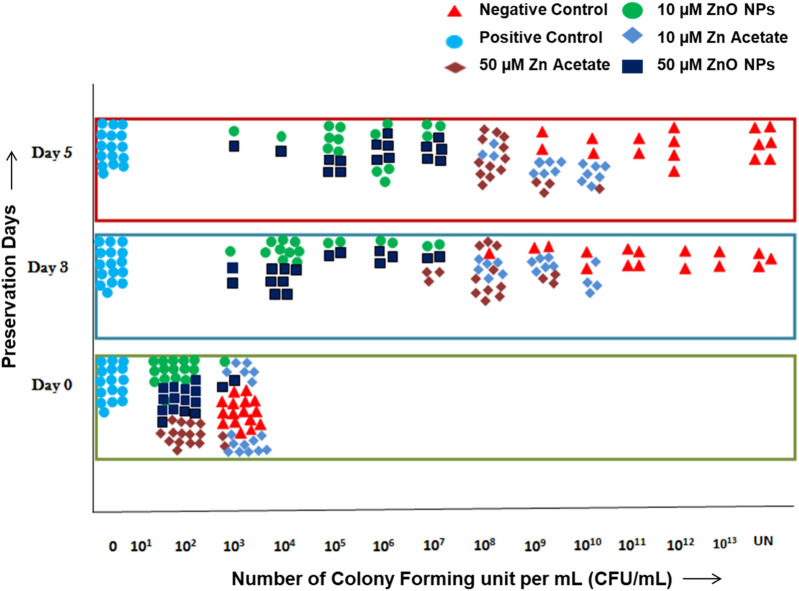
Antimicrobial test of semen extended in MODENA extender with ZnO-NPs, ZA salt, and penicillin–streptomycin antibiotic (positive control) and without antibiotic and NPs (negative control) at days 0, 3, and 5.

The antimicrobial activity of ZnO-NPs and ZA salt when compared to the positive control and negative control at days 0, 3, and 5 is presented (Figure 7; Supplementary Table S4). Bacterial growth at day 0 was 0 CFU/mL for the positive control, whereas it was 10^2^ CFUs/mL for ZnO-NPs at 10 µM and 50 µM and ZA at 50 µM, which was less compared to 10^3^ CFUs/mL for the negative control and ZA at 10 µM. Similarly, at day 3, growth was 10^3^–10^7^ CFUs/mL for ZnO-NPs (10µM, 50 µM) compared to the negative control with 10^8^ (hundred million)–uncountable CFUs/mL and other treatments such as ZA (50 µM) of 10^8^–10^9^ CFUs/mL and ZA (10 µM) of 10^8^–10^10^ CFUs/mL, but the positive control showed controlled growth due to the antibiotic effect. At day 5, the bacterial growth remained unchanged for ZnO-NPs (10µM and 50 µM) and ZA (50 µM and 10 µM) treatments from that of day 3 when compared to the negative control with an increase in colony count ranging from 10^9^ (one billion)–uncountable CFUs/mL. Meanwhile, the positive control continued to control the bacterial growth.

## 4 Discussion

In a nanoscale form, different metal and metal oxide NPs have exhibited new and innovative properties in biological and biomedical applications. In this context, the multifaceted role of ZnO-NPs is extensively documented, highlighting their attributes as a micronutrient, antimicrobial agent, antioxidative agent, and photocatalytic candidate and their efficacy as a facilitator in drug delivery and gas-sensing applications (Jiang et al., 2018; Mehnath et al., 2021). The application of ZnO-NPs in sperm biology, especially for sperm preservation and protection, is a novel addition. Furthermore, the utilization of ZnO-NPs in sperm biology research employing boar models enhances livestock production through AI, contributing not only to advancements in human andrology research but also to the improvement in animal breeding practices.

In the current evaluation, ZnO-NPs were synthesized and encapsulated using green material. The characterization of the synthesized ZnO-NPs was carried out with high-throughput (HTP) instruments deciphering the patterns of synthesis and nature of Zn in nano-form. Previous researchers also reported the development of ZnO-NPs through the green method (Jan et al., 2020).

The present study showed that a safe dose of ZnO-NPs was needed to avoid damage to the cells and their properties. This study observed no adverse effects at low concentrations of ZnO-NPs (5 μM, 10 μM, and 50 μM) in Sp-TALP media after 1 h of incubation at 37°C on sperm motility and functional attributes compared to the higher dose of NPs (≥100 μM). A similar report showed a high and significant cell death rate in human spermatozoa at higher doses of Zn-NPs (Barkhordari et al., 2013). It was proposed that the decline in semen quality resulting from higher doses of ZnO-NPs could be attributed to the interplay of reactive oxygen species (ROS) formation and the consequential reduction in MMP, ultimately culminating in an elevated incidence of apoptosis accompanied by nuclear DNA damage (Shen et al., 2019). The role of Zn-NPs as a potential drug in inducing apoptosis in various types of cancer cells has been extensively studied (Jiang et al., 2018; Shamasi et al., 2021). This particular property of Zn-NPs could be the reason why a noticeable increase in the levels of apoptotic and necrotic cells was observed at high concentrations of ZnO-NPs. Therefore, this study considered 5 μM, 10 μM, and 50 μM of ZnO-NPs as the non-toxic doses for boar sperm cells, and they were further selected to examine the effect on the boar sperm functional parameters during their short-term liquid preservation at 16°C.

Boar sperm can undergo several undesirable changes during preservation at 16°C, which can significantly reduce sperm quality and fertility (Bucak et al., 2010). NPs have demonstrated beneficial effects on semen preservation, and several metallic nanoparticles such as zinc (Jahanbin et al., 2021; Abedin et al., 2023a), selenium (Khalil et al., 2019), and silver have been shown to improve the quality of preserved semen along with the reduction of microbial load (Pérez-Duran et al., 2020). Furthermore, iron (Fe) NPs have been reported to aid in the purification of dead or damaged cells (Bisla et al., 2020). From our reports, it can be seen that ZnO-NPs at 10 μM, when supplemented with MODENA extender for extending boar semen, significantly increased sperm progressive motility, viability, membrane integrity, and MMP and reduced MDA levels compared to the control during preservation at 16°C for all days (day 0, day 3, and day 5). As per our literature search, there were no documented findings regarding the influence of ZnO-NPs on sperm motility in liquid-preserved boar semen. However, reports on Fe NPs showed improvement in the sperm linear motility of liquid-preserved boar semen after a 30-min exposure prior to storage (Tsakmakidis et al., 2020). The supplementation of ZnO-NPs in SHOTOR extender significantly improved the sperm progressive motility, viability, and membrane integrity of epididymal spermatozoa stored at 4°C (day 0, 1, 4, and 6) in dromedary camels (Shahin et al., 2020). Cryopreservation studies on bull and ram semen showed that zinc supplementation improved the MMP of post-thaw semen (Heidari et al., 2018; Jahanbin et al., 2021). It was also observed that Zn-NPs significantly reduced the level of MDA in post-thaw semen during cryopreservation of bull semen. Likewise, in human semen, Zn-NPs demonstrated a significant reduction in the level of MDA in post-thaw semen (Isaac et al., 2017). It was known that sperm cells possessed high levels of unsaturated fatty acids in their membranes and lacked essential cytoplasmic components to counter LPO and oxidative stress (Aitken et al., 1989; Storey, 1997; Bansal and Bilaspuri (2010). Therefore, our proposition posits that ZnO-NPs exhibit effective free-radical scavenging capabilities, with a well-established function in radical scavenging, as supported by prior *in vivo* and *in vitro* studies (Gualtieri et al., 2014; Afifi et al., 2015). Reports stated that Zn-NPs did not permeate viable cells and were localized on the surface of viable cells like a protective layer around the spermatozoa (Taylor et al., 2015; Abedin et al., 2023b). The current investigation additionally demonstrates the membrane-protective attributes of ZnO-NPs. This observation leads to the inference that ZnO-NPs play a role in averting membrane peroxidation, thereby contributing to the reduction of oxidative stress (Sanjay et al., 2014; Isaac et al., 2017). This may suggest ZnO-NPs’ protective function on membrane peroxidation and mitochondrial damage, thus preventing sperm cell death or apoptosis.

The present study revealed a significant increase in conception rates following AI upon supplementation with 10 μM ZnO-NPs compared to the control group during the preservation process. Several reports suggested that sperm motility and other parameters were correlated to the conception rate (Gadea, 2005; Nerin et al., 2014). Thus, the improved conception rate in our study was a direct result of ZnO-NPs’ positive effect on semen motility, viability, membrane integrity, and MMP for all days (days 0, 3, and 5) during liquid preservation at the non-cytotoxic doses.

AI is a common breeding technology in animals that requires the addition of antibiotics to semen extenders in its standard procedure to control microbial growth during preservation. Strepto-penicillin and gentamycin were added to control the prevalent bacterial contamination from the male reproductive tract and, to an extent, from the environment during preservation. Many researchers have been working on replacing antibiotics with other agents due to the increasing antibiotic-resistant microorganisms in the food chain. In the animal breeding industry, different replacing agents were tested ranging from antimicrobial alternatives such as peptides, poly extracts, or NPs to other physical methods of removal of bacteria (single-layer centrifugation and microfiltration) (Joerger, 2003; Morrell and Wallgren, 2011). However, disadvantages such as cost, sperm cell damage, and non-efficient antimicrobials led to the need to discover new and efficient antimicrobial alternatives. Previous studies reported that semen extender and storage duration have a significant effect on bacterial colonies (Luther et al., 2021). The current study showed that the antimicrobial activity of ZnO-NPs and ZA (salt control) at the selected non-cytotoxic doses in comparison to the negative control was in the order shown: the antimicrobial effect of ZnO-NP (10 µM) = ZnO-NP (50 µM) > ZA (50 µM) > ZA (10 µM) for day 0, 3, and 5. The study demonstrated that ZnO-NPs possessed an antibacterial property and reduced colony formation without affecting sperm cells. At day 0, ZnO-NP (10µM, 50 µM) treatment and ZA (50 µM) reduced the number of colonies formed compared to ZA (10 µM) and the negative control by 10-fold. At day 3, although the colonies formed increased due to the favorable temperature while preserving semen, ZnO-NPs at 10µM and 50 µM controlled bacterial growth up to 10^3^–10^7^ CFUs/mL followed by ZA at 50 µM of 10^8^–10^9^ CFUs/mL and ZA 10 µM of 10^8^–10^10^ CFUs/mL compared to the negative control with 10^8^ (hundred million)–uncountable CFUs/mL. At day 5, ZnO-NPs and ZA showed no increase in colony count, but it remained the same as that observed on day 3, hence controlling and inhibiting bacterial growth, but the negative control showed a high increase in colony count. The positive control showed controlled growth for days 0, 3, and 5, due to the antibiotic effect. Comparing ZnO-NPs (10 µM and 50 µM) and ZA salt (10 µM and 50 µM) to the negative control, one could infer the antibacterial activity of both ZnO-NPs and ZA when supplemented with MODENA extender, with the NPs exhibiting more antibacterial effect than the salt control. ZnO-NPs at 10 µM and 50 µM doses showed similar antibacterial activity. Thus, the ZnO-NP-supplemented MODENA extender acted as an antimicrobial active semen extender possessing an intrinsic antimicrobial capability against bacteria, allowing the reduction in the use of antibiotics against the standard antibiotic concentration without compromising antimicrobial activity and sperm quality (Luther et al., 2021). Such addition could reduce antibiotic usage in pig insemination and can provide a solution to the challenges experienced in the pig-rearing sector; such as the high semen storage temperature (15ºC–18°C) favoring bacterial growth, larger volume of insemination (50–100 mL), which generally requires two to three doses, and the possibility of increasing the number of resistance-rich genes in the soil due to backflow into manure of most insemination processes.

## 5 Conclusion

In conclusion, ZnO-NPs at low concentrations (5 μM, 10 μM, and 50 µM) demonstrated no cytotoxic effects on boar sperm, preserving its quality compared to higher concentrations. We recommend adding 10 µM ZnO-NPs in MODENA extender for improved sperm quality and conception rates after AI. Additionally, ZnO-NPs also showed antibacterial activity, suggesting a potential reduction in antibiotic use and resistance genes in the soil biome during the process of insemination. Further research should explore the dose and time-dependent toxicity of NPs in freezing protocols for diverse species, especially focusing on their impact on fertilization and early embryonic development.

## Data Availability

The original contributions presented in the study are included in the article/Supplementary Material; further inquiries can be directed to the corresponding author.

## Abbreviations

AI, artificial insemination; CFDA, carboxyfluorescein diacetate; CFU, colony forming unit; MMP, mitochondrial membrane potential; MDA, malonaldehyde; PI, propidium iodide; Sp-TALP, Sperm Tyrode’s albumin lactate pyruvate; and ZnO-NP, zinc oxide nanoparticle.
