# Supplementary material for: Synthesis of green zinc‐oxide nanoparticles and its dose‐dependent beneficial effect on spermatozoa during preservation: sperm functional integrity, fertility and antimicrobial activity
Source: Front Bioeng Biotechnol. 2024 Feb 23;12:1326143. doi: 10.3389/fbioe.2024.1326143 (PMC10920225; doi:10.3389/fbioe.2024.1326143)
Supplement: Supplementary file 1 [file Table1.DOCX]

**Supplementary data**

For the **figure 4, 5, 6 and 7**, supplementing data were provided below

Table 1: Effect of ZnO-NPs and Zinc Acetate on sperm progressive motility during short-term liquid preservation of boar semen

| **Group** | **Concentration (μM)** | | | **Concentration (μM)** | | | **Concentration (μM)** | | |
| --- | --- | --- | --- | --- | --- | --- | --- | --- | --- |
|  | **Day 0** | | | **Day 3** | | | **Day 5** | | |
|  | **5 µM** | **10 µM** | **50 µM** | **5 µM** | **10 µM** | **50 µM** | **5 µM** | **10 µM** | **50 µM** |
| **ZnO-NPs** | 89.42 ±0.91^a^ | 91 ±0.81^a^ | 90.2 ±0.82^a^ | 83.6± 1.03^abc^ | 87.6 ±0.85^a^* | 84.2 ±0.94^ab^* | 77.6 ±1.02^bc^ | 83.2 ±0.93^a^* | 80 ±0.91^ab^* |
| **ZA** | 89.6 ±0.87^a^ | 90.2 ±0.82^a^ | 89.4 ±0.91^a^ | 83.6 ±0.86^abc^ | 83 ±0.86^abc^ | 81.02 ±1.85^bc^ | 78.2 ±1.02^bc^ | 78.6 ±1.06^b^* | 76.4 ±1.11^bc^ |
| **Control** | 89 ±0.95^a^ | | | 79.2 ±0.99^c^ | | | 73.4 ±1.16^c^ | | |

Data shown as Mean ± SE, Values with different superscript in each day differ significantly (p≤0.05);

*Significant (p<0.05)

| **Table 2.**  **Effect of ZnO-NPs and Zinc Acetate** **on** **sperm viability and membrane integrity during short-term liquid preservation of boar semen.** | | | | | | | | | | |
| --- | --- | --- | --- | --- | --- | --- | --- | --- | --- | --- |
| **Group** | **Concentration (μM)** | | | **Concentration (μM)** | | | **Concentration (μM)** | | |  |
|  | **Day 0** | | | **Day 3** | | | **Day 5** | | |  |
|  | **5** | **10** | **50** | **5** | **10** | **50** | **5** | **10** | **50** |  |
| **ZnO-NPs** | 79.95±  1.04**^ab*^** | 82.52±  0.95**^a*^** | 79.98±  1.00**^ab*^** | 74.54 ±1.21**^ab*^** | 76.80 ±1.04**^a*^** | 74.25 ±1.15**^ab*^** | 69.58 ±1.39**^ab*^** | 72.06 ±1.26**^a*^** | 69.22±  1.34**^ab*^** |  |
| **ZA** | 77.86±  1.05**^bc^** | 78.28 ±0.99**^bc^** | 76.96 ±0.96**^bc^** | 72.84 ±1.23**^bc^** | 72.20 ±1.07**^bc^** | 71.33 ±1.11**^bc^** | 66.84 ±1.22**^abc^** | 66.72 ±1.35**^abc^** | 65.86 ±1.16**^bc^** |  |
| **Control** | 76.01 ±0.99**^c^** | | | 70.31 ±1.12**^c^** | | | 65.20 ±1.34**^c^** | | |  |
| Data shown as Mean ± SE, Values with different superscript in each day differ significantly (p≤0.05) *Significant (p<0.05)   \| **Table 3. Effect of ZnO-NPs and Zinc Acetate** **on** **mitochondrial membrane potential (MMP) during short-term liquid preservation of boar semen.** \| \| \| \| \| \| \| \| \| \| \| \| --- \| --- \| --- \| --- \| --- \| --- \| --- \| --- \| --- \| --- \| --- \| \|  \| **Group** \| **Concentration (μM)** \| \| \| **Concentration (μM)** \| \| \| **Concentration (μM)** \| \| \| \| **Day 0** \| \| \| **Day 3** \| \| \| **Day 5** \| \| \| \| **5** \| **10** \| **50** \| **5** \| **10** \| **50** \| **5** \| **10** \| **50** \| \| **ZnO-NPs** \| 78.69 ±0.84**^abc^** \| 81.69 ±0.83**^a*^** \| 79.90 ±0.83**^ab^** \| 71.25 ±0.76**^b^** \| 74.00 ±0.96**^a*^** \| 72.00 ±0.88**^ab^** \| 64.80 ±0.91**^abc^** \| 67.83 ±0.88**^a*^** \| 66.23 ±0.96**^ab*^** \| \| **ZA** \| 77.38 ±0.81**^bc^** \| 77.88 ±0.72**^bc^** \| 76.60 ±0.53**^c^** \| 71.26 ±0.67**^b^** \| 70.71 ±0.89**^b^** \| 70.14 ±0.66**^b^** \| 63.29 ±0.78**^bc^** \| 62.89 ±0.85**^bc^** \| 63.59 ±0.58**^bc^** \| \| **Control** \| 77.98 ±0.84**^bc^** \| \| \| 70.50 ±0.78**^b^** \| \| \| 62.19 ±0.68**^c^** \| \| \| \| Data shown as Mean ± SE, Values with different superscript in each day differ significantly (p≤0.05) *Significant (p<0.05) \| \| \| \| \| \| \| \| \| \| \| | | | | | | | | | | |

**Table 4. Antimicrobial activity (CFU/mL) of zinc-oxide nanoparticles on boar semen during short-term liquid preservation.**

| **Group** | **Concentration (μM)** | | **P^#^** | **Concentration (μM)** | | **p^#^** | **Concentration (μM)** | | **p^#^** |
| --- | --- | --- | --- | --- | --- | --- | --- | --- | --- |
|  | **Day 0** | |  | **Day 3** | |  | **Day 5** | |  |
|  | **10 μM** | **50 μM** |  | **10 μM** | **50 μM** |  | **10 μM** | **50 μM** |  |
| **ZnO-NPs** | 821.88^b^  ±385.65 | 919.38^b^  ±782.8 | **>0.05** | 51352745±  465288.64 | 1830021±  123513 | **<0.001** | 74457163^a^ ±54252691 | 336641950^a^ ±221207561 | **<0.001** |
| **ZA** | 2812.45±584.32**^b^** | 1932±485.65**^b^** | **<0.001** | 431574566±  1275311.29 | 429718292±  233153.29 | **>0.05** | 661744256±  2097156.31 | 713327587±  4103110.64 | **>0.05** |
| **Positive Control (with antibiotic)** | 0.00 | |  | 0.00 | |  | 0.00 | |  |
| **Negative Control (without antibiotic)** | 5658.38 ±2549.12**^b^** | |  | 2033860500 ±130509786**^a^** | |  | TMTC | |  |
| **p^*^** | **<0.001** | |  | **<0.001** | |  | **<0.001** | |  |
| Data shown as Mean ± SE  p^*^- ANOVA for repeated measures.; P^#^ - t- test  Values with different superscript (a, b) in each day differ significantly (p<0.05)  TMTC- Too much to count | | | | | | | | | |
