# Supplementary figures and images for: Synthesis of green zinc‐oxide nanoparticles and its dose‐dependent beneficial effect on spermatozoa during preservation: sperm functional integrity, fertility and antimicrobial activity
Source: Front Bioeng Biotechnol. 2024 Feb 23;12:1326143. doi: 10.3389/fbioe.2024.1326143 (PMC10920225; doi:10.3389/fbioe.2024.1326143)

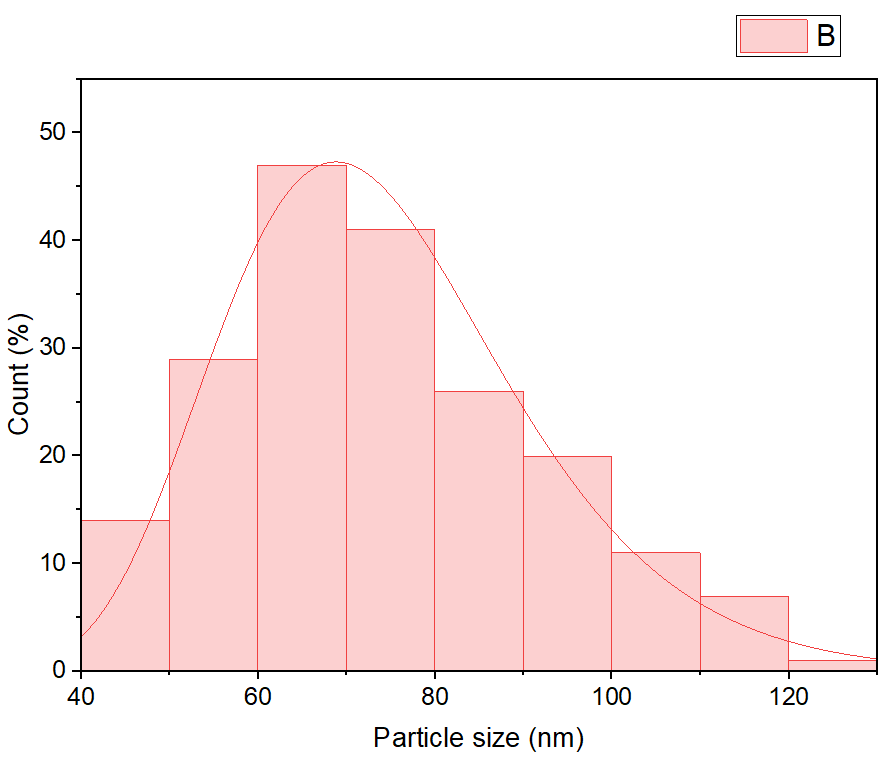

Supplement: Supplementary file 2 [file Image1.PNG]
